# Incidence Rates of COVID-19-Associated Hospitalization and Risk Factors for Severe Disease Among American Indian and Alaska Native Persons in the Southwest USA and Alaska

**DOI:** 10.1007/s40615-025-02492-9

**Published:** 2025-07-21

**Authors:** Chelsea S. Lutz, Catherine G. Sutcliffe, James W. Keck, Rachel M. Hartman, Christine Desnoyers, Amy Swango-Wilson, Amanda B. Burrage, Angela P. Campbell, Loretta Christensen, Ryan M. Close, Shawnell Damon, Jennifer Dobson, Starla Garcia, Natasha Halasa, Elvira Honie, Verlena Little, Meredith L. McMorrow, Dennie Parker, Mila M. Prill, Jennifer Richards, Puthiery Va, Mark Veazie, Dan VanDeRiet, Del Yazzie, Rosalyn J. Singleton, Laura L. Hammitt

**Affiliations:** 1 Department of International Health, Johns Hopkins Bloomberg School of Public Health, Baltimore, MD USA; 2 Department of Epidemiology, Johns Hopkins Bloomberg School of Public Health, Baltimore, MD USA; 3 Alaska Native Tribal Health Consortium, Anchorage, AK USA; 4 Yukon-Kuskokwim Health Corporation, Bethel, AK USA; 5 Tuba City Regional Health Care Corporation, Tuba City, AZ USA; 6 Coronavirus and Other Respiratory Viruses Division, Centers for Disease Control and Prevention, Atlanta, GA USA; 7 Indian Health Service, Rockville, MD USA; 8 Whiteriver Service Unit, Phoenix Area Indian Health Service, Whiteriver, AZ USA; 9 Navajo Area Indian Health Service, St. Michaels, AZ USA; 10 Vanderbilt University Medical Center, Nashville, TN USA; 11 Chinle Service Unit, Navajo Area, Indian Health Service, Chinle, AZ USA; 12 Navajo Epidemiology Center, Window Rock, AZ USA

**Keywords:** COVID-19, SARS-CoV-2, Indigenous health, Risk factor

## Abstract

**Introduction:**

COVID-19 causes significant morbidity in the USA, particularly among American Indian/Alaska Native (AI/AN) persons. Estimates of COVID-19 burden among AI/AN communities are needed to identify health outcome disparities and inform prevention strategies, but under-ascertainment of AI/AN status in national data may result in underestimation of COVID-19 disease burden.

**Methods:**

Surveillance for acute respiratory illness was conducted among AI/AN persons at eight healthcare facilities in Arizona and Alaska to identify COVID-19-associated hospitalizations and outpatient visits. Weekly and annual incidence rates of COVID-19-associated hospitalizations per 100,000 persons were calculated overall and by site and age. Risk factors for COVID-19-associated hospitalizations (versus outpatient visits) were assessed.

**Results:**

From January 2021 to December 2022, 1159 COVID-19-associated hospitalizations were identified. Incidence rates were 439.8 per 100,000 in 2021 and 332.6 per 100,000 in 2022 and highest among adults ≥ 65 years at all sites. Compared to national estimates from 2021 to 2022, incidence rates by time and age were similar among older adults, whereas incidence rates among AI/AN children were over twice as high. Among adults, older age, chronic lung disease, chronic kidney disease, and diabetes increased the risk of hospitalization; frequent mask use outside the home and COVID-19 vaccination were protective, particularly if vaccinated within the past year. Among children, younger age and heart conditions increased the risk of hospitalization.

**Conclusions:**

The findings demonstrate a substantial burden of COVID-19 in AI/AN persons and provide critically needed data regarding the risks for severe outcomes. AI/AN children experience a disproportionate burden of COVID-19 disease.

**Supplementary Information:**

The online version contains supplementary material available at 10.1007/s40615-025-02492-9.

## Introduction

COVID-19 continues to cause a significant disease burden worldwide and has resulted in over 7 million deaths globally since December 2019 [1]. Known risk factors for severe COVID-19 include chronic kidney, liver, and lung disease, diabetes, heart conditions, and obesity, among others [2]. Such conditions disproportionately affect Indigenous persons compared to other racial/ethnic groups in the USA [3–5]. In addition, relative to other racial/ethnic groups, Indigenous persons — especially the estimated 1.26 million American Indian/Alaska Native (AI/AN) persons living in tribal lands [6] — experience higher rates of poverty and greater challenges accessing healthcare services, putting them at higher risk of poor health outcomes [7–10]. Data collected through April 19, 2023, demonstrated that when adjusted for age, AI/AN persons are 2.4 times more likely to be hospitalized and 2.0 times more likely to die because of COVID-19 than non-Hispanic White Americans [11]. Misclassification of AI/AN persons as White or “other,” as well as inconsistent reporting of race/ethnicity in national COVID-19 data overall, has likely resulted in the underestimation of the true COVID-19 burden among AI/AN persons and communities [12–14].

Data on COVID-19 hospitalization and risk factors for severe disease among AI/AN persons are needed to improve understanding of health outcome disparities and inform disease prevention strategies. Risk factors for severe COVID-19 have been documented extensively in the literature [15–20]. However, representation of AI/AN persons in these observational studies has been limited. We conducted respiratory disease surveillance among AI/AN communities in Alaska (AK) and Arizona (AZ) to estimate incidence rates of COVID-19-associated hospitalizations and identify risk factors for severe COVID-19 in AI/AN persons.

## Methods

### Study Design and Population

Active surveillance for acute respiratory illness (ARI) was conducted among AI/AN persons at eight healthcare facilities in Arizona (Chinle and Tuba City in the Navajo Nation, Whiteriver in the White Mountain Apache Tribal lands) and Alaska (the Anchorage municipality and 58 federally recognized tribes in the Yukon-Kuskokwim [YK] Delta in western Alaska), as previously described [21]. Briefly, eligible persons (Supplemental Table 1) of all ages presenting for inpatient or outpatient care at participating Indian Health Service and/or Tribal Health facilities (Supplemental Table 2) were enrolled after obtaining parental permission or informed consent, as appropriate. Study staff conducted inpatient surveillance and enrollment 5–6 days per week, ensuring no more than one consecutive day without on-site monitoring; inpatient admissions records from days when staff were not on site were reviewed the next day and any potential participants admitted during that time were contacted for enrollment at the earliest opportunity. All potentially eligible inpatient individuals were documented, including those discharged or transferred before study staff could make contact. Arizona sites also enrolled participants from outpatient facilities 2–4 days per week. Enrollment at the Anchorage, Chinle, YK Delta, and Whiteriver sites occurred from January 1, 2021, to December 31, 2022; enrollment at the Tuba City site occurred from May 17, 2021, to December 31, 2022. The incidence rate calculations accounted for all eligible individuals (both enrolled and non-enrolled), whereas only enrolled individuals were included in the risk factor analyses.

### Data Collection

Information on age, sex, SARS-CoV-2 clinical testing, and presenting symptomology were recorded for all eligible patients. Following informed consent, information on enrolled participants’ sociodemographic and household characteristics, self-reported symptoms, previous medical care, self-reported COVID-19 vaccination status, and mask use was collected via interview. Mask use was assessed using the question “When you/your child leave the home (e.g., to go to work or the grocery store/to visit friends or go to school), do you/does your child wear a mask or face covering?” with the following answer choices: never, sometimes, usually, or always. Mask use was assessed to account for potential reduction in viral particles a person may be exposed to, as well as a proxy for general risk-reducing behaviors. Electronic health records (EHR) were reviewed to collect information regarding underlying medical conditions, clinical testing for SARS-CoV-2 (including date, test type, and results), and clinical course and outcomes of the current illness. COVID-19 vaccination status was also ascertained from EHR and state vaccine registries. Discrepancies between EHR-documented and self-reported vaccination status were verified, including re-contacting participants if appropriate; we deferred to dates from EHR/state registries unless these were missing.

Mid-turbinate nasal swab research specimens or residual clinical nasal specimens were collected from every enrolled participant. Specimens were sent to Vanderbilt University Medical Center (VUMC) for surveillance testing via singleplex polymerase chain reaction (PCR) for SARS-CoV-2. Testing for SARS-CoV-2 and interpretation of results were performed according to methods described in the CDC Emergency Use Authorization protocol [22].

### Case Definitions

#### COVID-19-Associated Hospitalization

Persons who presented to the healthcare facility, met the eligibility criteria, and enrolled in the study were considered to have a COVID-19-associated hospitalization if a SARS-CoV-2-positive test result occurred during hospitalization or during the 14 days preceding hospitalization. Results from clinical and research testing for SARS-CoV-2 were used for the analysis. If a positive SARS-CoV-2 test occurred more than 14 days prior to the hospitalization, we considered those test results as not associated with the hospitalization. Reinfections were counted as new incident infections and considered in this analysis when associated with a COVID-19 hospitalization as defined above. Reinfections were defined as a positive SARS-CoV-2 test that occurred at least 90 days after the initial case-defining test. Hospitalizations with a positive SARS-CoV-2 test result that occurred less than 90 days after a COVID-19-associated hospitalization were excluded from all analyses.

#### Severe COVID-19-Associated Hospitalization

Severe COVID-19-associated hospitalization was defined among participants with COVID-19-associated hospitalization according to the World Health Organization’s definition and required at least one of the following during the course of illness: use of supplemental oxygen by non-invasive ventilation or high-flow nasal cannula, intubation or mechanical ventilation, use of vasopressors, dialysis, extracorporeal membrane oxygenation (ECMO), or death [23].

#### Outpatient COVID-19 Illness

Outpatient COVID-19 illness was defined if a SARS-CoV-2-positive test result occurred during the medical visit to an outpatient facility or emergency department or during the 14 days preceding the visit.

All of the above medical visits were defined as COVID-19-associated if ≥ 1 diagnostic test from a participant’s visit had a positive result for SARS-CoV-2. Because of difficulty accessing patient rooms and safety considerations during the COVID-19 pandemic, the collection of research swabs was often delayed relative to participants’ presentation for care. Therefore, case status was maintained if the participant had a positive clinical molecular or antigen test, but the research result was negative.

### Data Analysis

#### Incidence Rates

Age-specific incidence rates per 100,000 persons were calculated as the number of COVID-19-associated hospitalizations divided by the number of persons in the Indian Health Service (IHS) User Population (i.e., all AI/AN people who received IHS-funded health care services at least once at the facility during the preceding three years) in each age group, multiplied by 100,000. Numerators (i.e., cases) were weighted to account for the proportion of hospitalized persons for whom SARS-CoV-2 test results within the 14-day window were not available. This was done by applying the proportion of SARS-CoV-2-positive results among the total tested to the total number screened and hospitalized with ARI, with site, age group (0–4 years, 5–17 years, 18–49 years, 50–64 years, ≥ 65 years, all ages), and time frame (weekly and annually) as sampling strata. Weekly and moving 3-week average incidence rates for the entire study period, defined according to epidemiologic week [24], and overall annual incidence rates for 2021 and 2022 were calculated. The denominator for 2021 in Tuba City was annualized to adjust for 33 weeks of surveillance. Generalized linear models assuming a Poisson distribution were used to calculate incidence rates and 95% confidence intervals (CIs). Where there were zero hospitalizations, 95% CIs were calculated using exact methods. Annual age-specific incidence rates were compared to national estimates from the COVID-NET interactive dashboard for 2021 and 2022 (Table 2) by calculating the ratio of the incidence estimates for each age group.

#### Risk Factor Analysis

Risk factors for any COVID-19-associated hospitalization were evaluated by comparing characteristics of participants with COVID-19-associated hospitalization versus COVID-19 outpatient illness. Analyses were stratified by age group (i.e., adults ≥ 18 years and children < 18 years). Covariates of interest included age, sex, enrollment site, education level, access to running water in home, use of wood to heat home, underlying medical conditions, vaccination status, frequency of mask use, and number of days between illness onset and care seeking, which were defined using participant questionnaires and chart review data. Continuous variables were expressed as medians and interquartile ranges. Categorical variables were summarized as counts and percentages. Bivariable and multivariable analyses were conducted using Poisson regression models with clustered standard errors (to account for reinfections) to estimate risk ratios (RR) and 95% CIs. Variables that were statistically significant in bivariable analysis were considered for inclusion in multivariable models. Potential covariates were assessed for collinearity using variance inflation factors (VIFs) and condition number metrics: VIFs ≤ 5 and condition numbers ≤ 10 were considered acceptable for inclusion. The Wald test was used to assess the statistical significance of coefficient effects, with significance determined at *p* < 0.05. Missing covariates were noted and a cutoff of > 10% missingness was used to exclude covariates from the multivariable analysis.

The risk factor analysis in adults was restricted to participants enrolled from the Chinle and Tuba City sites because Alaska sites only enrolled inpatient participants and over 90% of participants at the Whiteriver site were inpatients. Given the interest in vaccination status and the introduction of booster doses during the study period, separate models were built to evaluate the association between demographic and clinical covariates and COVID-19-associated hospitalization during the entire study period and the association between vaccination status and timing and COVID-19-associated hospitalization stratified by availability of COVID-19 booster doses. Booster availability was defined using Navajo Nation community announcements and was as follows: adults aged ≥ 65 years were eligible starting September 25, 2021; adults aged ≥ 18 with an underlying medical condition conferring greater risk for severe COVID-19 outcomes were eligible starting November 7, 2021; and all adults aged ≥ 18 were eligible starting November 10, 2021 [25–27]. An exploratory analysis for risk factors for severe COVID-19-associated hospitalization was also conducted using the same methods described for any COVID-19-associated hospitalization above. The small number of severe hospitalizations limited the number of covariates that could be evaluated in bivariable and multivariable analyses. As vaccination was considered a strong confounder, RRs for each covariate adjusted for vaccination status were estimated. The analysis in children was restricted to participants enrolled from the Chinle, Tuba City, and Whiteriver sites [28]. Given the small number of children included in the analysis who were vaccinated, the association between demographic, clinical, and vaccination covariates and COVID-19-associated hospitalization was evaluated during the entire study period. To evaluate potential sources of bias in the risk factor analyses, Pearson’s chi-square and Fisher’s exact tests were used to assess differences in select demographic characteristics among SARS-CoV-2-positive, eligible inpatients who were consented, excluded, or declined enrollment.

All statistical analyses were performed using SAS software, Version 9.4 (SAS Institute Inc, Cary, NC) and Stata/SE 17.0 (StataCorp LLC, College Station, TX).

## Results

### Eligibility Screening and Enrollment

From January 1, 2021, to December 31, 2022, 3665 eligible inpatient persons with ARI were identified. Among eligible inpatients, 1530 (41.7%) consented, 1159 (31.6%) declined, and 976 (26.6%) were unable to be consented (e.g., transferred to a different facility and unable to be contacted). Eighteen SARS-CoV-2-positive inpatients tested positive twice within a 90-day period, and their second event was excluded from the analysis, leaving 3647 inpatients including 1523 (41.8%) consented, 1154 (31.6%) declined, and 970 (26.6%) unable to be consented (Table 1). Seventeen inpatients tested positive for SARS-CoV-2 twice, more than 90 days apart, during the study period and were included in the analyses as reinfections. Among all eligible inpatients, 48.5% were female, 28.2% were < 18 years old, 29.6% were ≥ 65 years old, and 96.5% had an available SARS-CoV-2 test result, of whom 32.9% tested positive (Table 1). Among outpatients, 713 were consented; no outpatients tested positive more than once within a 90-day period (Table 1). One outpatient individual tested positive for SARS-CoV-2 twice, more than 90 days apart, during the study period and was included in the analyses as a reinfection.

**Table 1:** Characteristics of eligible potential American Indian/Alaska Native participants at participating facilities in Arizona and Alaska, January 1, 2021 – December 31, 2022

	Screened inpatients				Consented outpatients			
	Total eligible inpatients (*N* = 3647)	SARS-CoV-2 positive (*n* = 1159)	SARS-CoV-2 negative (*n* = 2359)	No test results (*n* = 129)	Total consented outpatients (*N* = 713)	SARS-CoV-2 positive (*n* = 209)	SARS-CoV-2 negative (*n* = 486)	No test results (*n* = 18)
	*n* (%)	*n* (%)	*n* (%)	*n* (%)	*n* (%)	*n* (%)	*n* (%)	*n* (%)
Age in years, median (IQR)	48.3 (6.5–68.7)	56.5 (37.3–71.8)	41.4 (2.6–66.0)	55.1 (24.4–72.2)	27.1 (4.9–46.8)	34.6 (12.9–51.2)	20.8 (4.5–45.7)	4.3 (2.2–5.4)
Age group in years								
0 to 4	854 (23.4)	109 (9.4)	724 (30.7)	21 (16.3)	181 (25.4)	27 (12.9)	141 (29.0)	13 (72.2)
5 to 17	176 (4.8)	26 (2.2)	142 (6.0)	8 (6.2)	136 (19.1)	39 (18.7)	94 (19.3)	3 (16.7)
18 to 49	861 (23.6)	339 (29.2)	493 (20.9)	29 (22.5)	252 (35.3)	84 (40.2)	166 (34.2)	2 (11.1)
50 to 64	677 (18.6)	267 (23.0)	385 (16.3)	25 (19.4)	99 (13.9)	37 (17.7)	62 (12.8)	36 (11.2)
≥ 65	1079 (29.6)	418 (36.1)	615 (26.1)	46 (35.7)	45 (6.3)	22 (10.5)	23 (4.7)	42 (13.0)
Sex								
Female	1768 (48.5)	507 (43.7)	1187 (50.3)	74 (57.4)	273 (38.3)	77 (36.8)	187 (38.5)	9 (50.0)
Male	1879 (51.5)	652 (56.3)	1172 (49.7)	55 (42.6)	440 (61.7)	132 (63.2)	299 (61.5)	9 (50.0)
Site								
Anchorage, AK	688 (18.9)	244 (21.1)	437 (18.5)	7 (5.4)	NA	NA	NA	NA
YK Delta, AK	714 (19.6)	151 (13.0)	548 (23.2)	15 (11.6)	NA	NA	NA	NA
Chinle, AZ	1035 (28.4)	335 (28.9)	654 (27.7)	46 (35.7)	332 (46.6)	75 (35.9)	251 (51.6)	6 (33.3)
Tuba City, AZ	459 (12.6)	164 (14.2)	250 (10.6)	45 (34.9)	251 (35.2)	120 (57.4)	125 (25.7)	6 (33.3)
Whiteriver, AZ	751 (20.6)	265 (22.9)	470 (19.9)	16 (12.4)	130 (18.2)	14 (6.7)	110 (22.6)	6 (33.3)
Disposition status								
Consent	1523 (41.8)	479 (41.3)	1038 (44.0)	6 (4.7)	713 (100.0)	209 (100.0)	486 (100.0)	18 (100.0)
Decline	1154 (31.6)	355 (30.6)	732 (31.0)	67 (51.9)	NA	NA	NA	NA
Non-enrolled	970 (26.6)	325 (28.0)	589 (25.0)	56 (43.4)	NA	NA	NA	NA

All eligible inpatients screened (i.e., regardless of study disposition) were included in COVID-19-associated hospitalization incidence rate calculations; only consented participants (outpatients and inpatients) from Southwest sites were included in risk factor analyses. Alaska sites did not enroll outpatients. Inpatients were screened 5–6 days per week and outpatients were screened 2–4 days per week at 6 inpatient facilities and 2 outpatient facilities serving American Indian and Alaska Native persons in Chinle, Tuba City, and Whiteriver, AZ; Anchorage and Yukon-Kuskokwim Delta, AK

*AK* Alaska, *AZ* Arizona, *IQR* interquartile range, *NA* not applicable

### COVID-19 Incidence

**Fig. 1:**
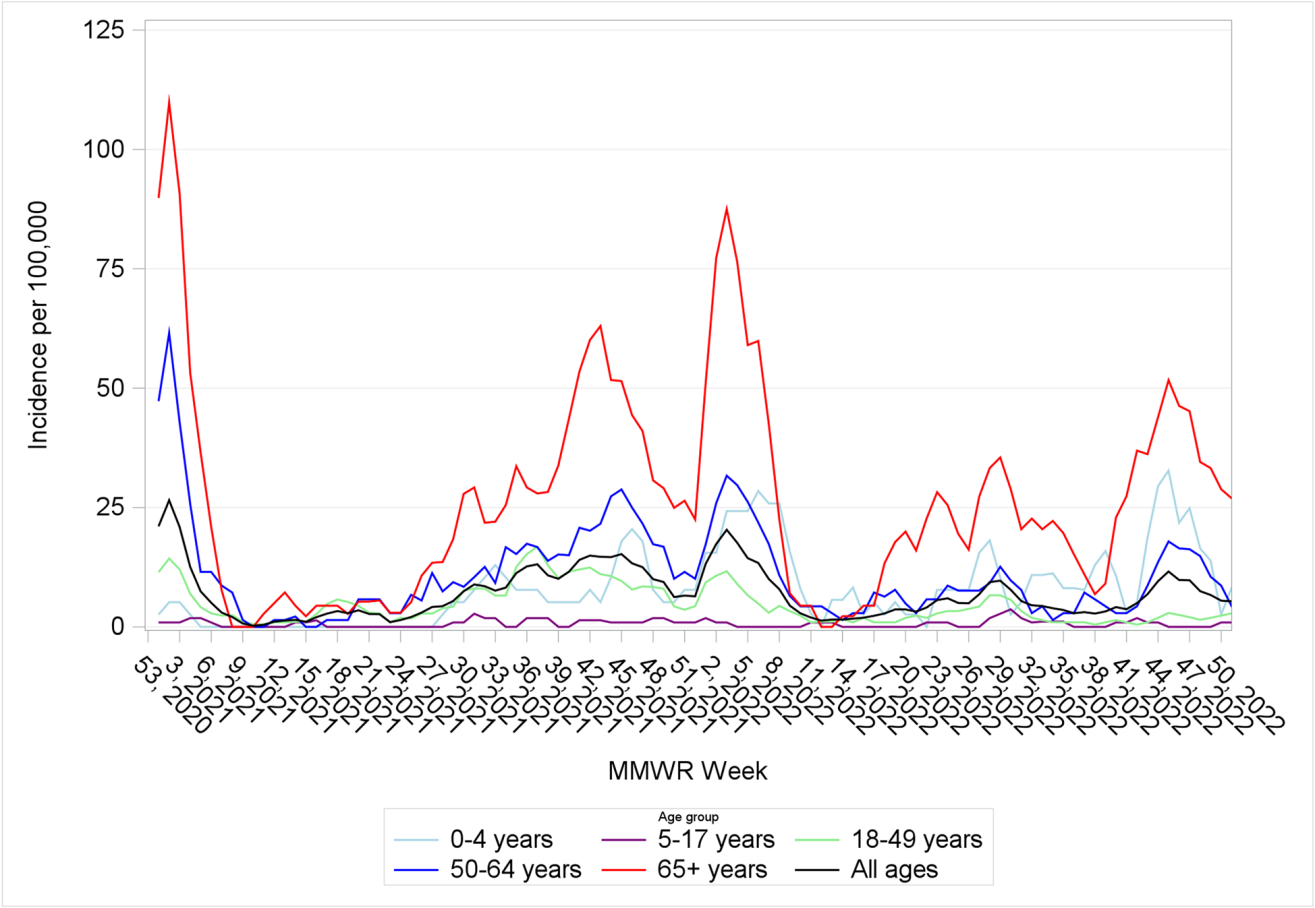
Weekly incidence rate (3-week moving average) of COVID-19-associated hospitalizations, by age group, among American Indian/Alaska Native persons at participating facilities in Arizona and Alaska, January 1, 2021–December 31, 2022. MMWR Week 53, 2020 corresponds to the week ending 01/02/2021; MMWR Week 52, 2021 corresponds to the week ending 01/01/2022; MMWR Week 52, 2022 corresponds to the week ending December 31, 2022. MMWR weeks start on Sunday and end on the following Saturday. Participating facilities include 6 Indian Health Service and Tribal Health facilities serving Navajo Nation (Chinle, Tuba City) and the White Mountain Apache (Whiteriver) Tribe in Arizona, and two Alaska Native medical centers serving AI/AN persons in Anchorage and the Yukon-Kuskokwim Delta, Alaska

Across all communities, adults aged ≥ 65 years had the highest weekly COVID-19-associated hospitalization incidence rates for most of the study period, followed by adults 50–64 years (Fig. 1). Weekly incidence rates peaked in January 2021, during fall 2021 and January 2022 coinciding with Delta and Omicron variant circulation, respectively, and again in November 2022. The moving 3-week average incidence of COVID-19-associated hospitalization by age group and community is presented in Supplemental Figs. 1–6. The largest weekly peaks were observed in Whiteriver, AZ, and the lowest in Anchorage, AK.

**Fig. 2:**
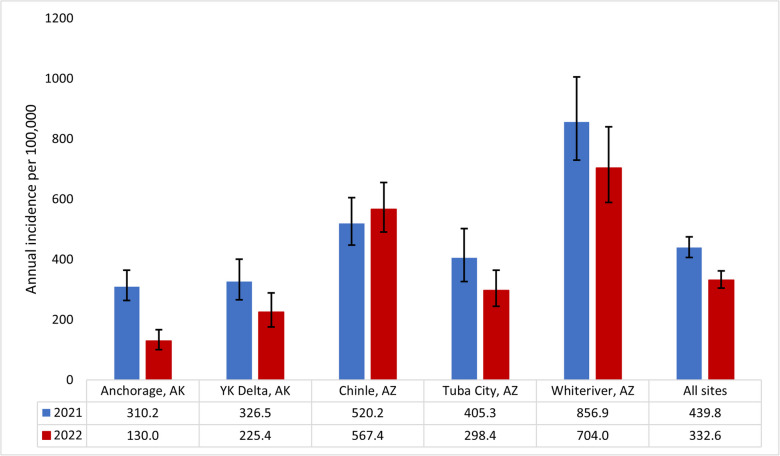
Annual incidence rates per 100,000 persons of COVID-19-associated hospitalizations among American Indian/Alaska Native persons at participating facilities in Arizona and Alaska, by enrollment site, January 1, 2021–December 31, 2022. AK: Alaska; YK: Yukon-Kuskokwim; AZ: Arizona. Tuba City 2021 denominator adjusted to account for 33 weeks of surveillance

Overall annual rates were higher in 2021 compared to 2022 across all sites except Chinle (Fig. 2); children aged 0–4 years were the only group for which 2022 rates were higher than 2021 rates across all sites except Anchorage (Supplemental Fig. 7). Annual incidence rates per 100,000 by age group and enrollment site are presented in Supplemental Figs. 7–11. In both years, incidence was highest among adults aged ≥ 65 years across all sites. In 2021, the next highest rates were estimated among adults 50–64 years. In 2022, the next highest rates varied across sites between adults 50–64 years and children 0–4 years. Incidence rates among adults were higher in the Southwest than Alaska particularly in 2022, but varied among children, with the highest rates among children 0–4 years observed in YK Delta in 2021.

Compared to national estimates, annual COVID-19-associated hospitalization incidence rates among children aged 0–4 years old were 2.3 and 2.7 higher among study participants (across all sites) compared to national estimates during 2021 and 2022, respectively (Table 2). Compared to older adults (50–64 and ≥ 65 years), 2021 rates among study participants were comparable to national estimates (Table 2). Conversely, during 2022, rates among study participants aged 5–17 and 18–49 years were approximately 40% lower than national estimates for each respective age group.

**Table 2:** Annual COVID-19-associated hospitalization incidence rate per 100,000 persons among study participants and according to United States national estimates, by age group, 2021 and 2022

	Study estimates		National estimates^a^		Ratio of study to national estimates	
	2021	2022	2021	2022	2021	2022
0–4 years	244.5	616.1	107.2	232.1	2.3	2.7
5–17 years	45.7	31.3	52.5	53.0	0.9	0.6
18–49 years	355.7	155.1	323.9	244.5	1.1	0.6
50–64 years	759.0	474.5	711.5	482.9	1.1	1.0
≥ 65 years	1536.8	1433.8	1230.0	1583.7	1.2	0.9

Tuba City 2021 denominator adjusted to account for 33 weeks of surveillance

^a^Estimated using data from CDC COVID-NET (https://www.cdc.gov/coronavirus/2019-ncov/your-health/covid-net.html). Weekly rates for each age group were summed by calendar year (January 2021-December 2021 and January 2022-December 2022), using the overall category for sites, race/ethnicity, and sex

### Risk Factor Analysis

Among 707 participants aged ≥ 18 years enrolled at the Chinle and Tuba City sites, 298 (42.1%) were SARS-CoV-2-positive, including 164 inpatients (55.0%) and 134 outpatients (45.0%). Median age was 52.0 years, 68.5% were female, and underlying conditions were common: 16.1% had asthma, 41.6% had diabetes, 31.9% had hypertension, and 29.2% had obesity (Table 3). Over the entire time frame, 187 (62.8%) participants completed a primary COVID-19 vaccine series prior to their illness onset; of adults who were vaccinated after COVID-19 booster doses were available (*n* = 132), 97 (73.5%) completed a primary series only and 35 (26.5%) received a primary series and at least one booster dose (Table 4). Twenty-one (12.8%) of 164 hospitalizations were classified as severe (Supplemental Tables 1 and 3). A comparison of consented, declined, and excluded eligible SARS-CoV-2-positive inpatients is provided in Supplemental Table 4. Consented inpatient participants were significantly more likely to be female and younger than SARS-CoV-2-postive patients who were excluded or declined enrollment.

**Table 3:** Participant characteristics and risk factors for COVID-19-associated hospitalization among American Indian/Alaska Native adults at participating facilities in Arizona, January 1, 2021 – December 31, 2022

	Outpatient (*n* = 134)	Hospitalization^a^ (*n* = 164)	Crude RR (95% CI)	Adjusted RR (95% CI)^b,c^
Age in years (median [IQR])	45.3 (33.0–60.3)	55.9 (43.0–67.5)	**1.01 (1.01–1.02)**	Not included
Age group in years				
18–49	78 (58.2)	55 (33.5)	REF 1.00	REF 1.00
50–64	34 (25.4)	62 (37.8)	**1.56 (1.21–2.02)**	**1.44 (1.13–1.85)**
≥ 65	22 (16.4)	47 (28.7)	**1.65 (1.27–2.14)**	**1.53 (1.16–2.01)**
Sex				
Male	36 (26.9)	58 (35.4)	REF 1.00	REF 1.00
Female	98 (73.1)	106 (64.6)	0.84 (0.68–1.04)	0.94 (0.77–1.15)
Medical condition or risk factor present				
Alcohol and/or substance abuse	12 (9.0)	20 (12.2)	1.15 (0.86–1.55)	Not included
Asthma	26 (19.4)	22 (13.4)	0.81 (0.58–1.12)	Not included
Chronic lung disease	1 (0.7)	6 (3.7)	**1.58 (1.15–2.18)**	**1.79 (1.07–3.01)**
Current or former smoker^d^	19 (14.2)	30 (18.3)	1.17 (0.91–1.52)	Not included
Chronic liver disease	7 (5.2)	9 (5.5)	1.02 (0.66–1.60)	Not included
Chronic kidney disease	2 (1.5)	17 (10.4)	**1.70 (1.40–2.06)**	**1.67 (1.26–2.21)**
Diabetes (type 1 or 2)	39 (29.1)	85 (51.8)	**1.51 (1.23–1.85)**	**1.36 (1.10–1.68)**
Heart condition	18 (13.4)	26 (15.9)	1.09 (0.83–1.42)	Not included
Hypertension	37 (27.6)	58 (35.4)	1.17 (0.95–1.44)	Not included
Immunocompromised	4 (3.0)	6 (3.7)	1.09 (0.65–1.84)	Not included
Mental health condition	18 (13.4)	22 (13.4)	1.00 (0.74–1.36)	Not included
Obesity	38 (28.4)	49 (29.9)	1.03 (0.83–1.29)	Not included
Supplemental oxygen use at home^d^	0	15 (9.1)	**1.92 (1.72–2.16)**	1.15 (0.80–1.64)
Running water in home^d^	108 (80.6)	117 (71.3)	0.88 (0.68–1.15)	Not included
Wood used to heat home	77 (57.5)	93 (56.7)	0.99 (0.80–1.22)	Not included
Education level^d^				
Some high school or less	15 (11.2)	19 (11.6)	Not included	Not included
High school diploma or GED	37 (27.6)	43 (26.2)	Not included	Not included
Some college or AA degree	56 (41.8)	63 (38.4)	Not included	Not included
College degree (incl. advanced)	20 (14.9)	5 (3.0)	Not included	Not included
Frequency of mask use outside of home^d,e^				
Never or sometimes	4 (3.0)	16 (9.8)	REF 1.00	REF 1.00
Usually or always	126 (94.0)	138 (84.1)	**0.65 (0.51–0.84)**	**0.63 (0.49–0.81)**
Days from illness onset to care seeking				
0–7	122 (91.0)	132 (80.5)	REF 1.00	REF 1.00
≥ 8	12 (9.0)	32 (19.5)	**1.40 (1.13–1.74)**	1.19 (0.92–1.55)

Reference category for all medical conditions or risk factors, running water in home, and wood used to heat home was “no.” Boldface indicates statistical significance of *p* < 0.05. Alaska sites only enrolled inpatients and over 90% of participants at the Whiteriver, AZ site were inpatients, so adult risk factor analysis restricted to Chinle and Tuba City sites only. Inpatients were screened 5–6 days per week and outpatients were screened 2–4 days per week

*AA* associates degree, *CI* 95% confidence interval, *GED* general educational development, *IQR* interquartile range, *NA* not applicable (i.e., zeros in cells), *REF* reference category, *RR* risk ratio

^a^Any COVID-19-hospitalization, includes those classified as severe

^b^Adjusted for age group, service unit (enrollment site), sex, presence of chronic lung disease, chronic kidney disease, diabetes (type 1 or 2), supplemental oxygen use at home, vaccination status, mask use, and number of days between illness onset and care seeking

^c^Vaccination status (unvaccinated, received primary series only, received primary series plus at least one booster dose) included in adjusted models; results for vaccination status are presented separately in Table 4. Completed primary series = received two doses of an approved mRNA COVID-19 vaccine primary series or one dose of an approved non-mRNA vaccine ≥ 14 days prior to illness onset

^d^Seventeen (5.7%) participants missing current or former smoking status, 6 (2.0%) missing supplemental oxygen use at home, 22 (7.4%) missing running water in home, 40 (13.4%) missing education level, 14 (4.7%) missing frequency of mask use outside of home. Variables with > 10% missingness were not included in univariable or multivariable models

^e^Mask use was assessed using the question “When you/your child leave the home (e.g., to go to work or the grocery store/to visit friends or go to school), do you/does your child wear a mask or face covering?” with the following answer choices: never, sometimes, usually, or always

**Table 4:** Association between vaccination status, timing of vaccination, and COVID-19-associated hospitalization, prior to and after availability of COVID-19 vaccine booster dose among American Indian/Alaska Native adults at participating facilities in Arizona, January 1, 2021–December 31, 2022

	Prior to availability of COVID-19 vaccine booster dose				After availability of COVID-19 vaccine booster dose			
	Outpatient (*n* = 61)	Any hospitalization (*n* = 54)	Crude RR (95% CI)	Adjusted RR^a^ (95% CI)	Outpatient (*n* = 73)	Any hospitalization (*n* = 110)	Crude RR (95% CI)	Adjusted RR^a^ (95% CI)
Vaccination status^b^								
Unvaccinated	16 (26.2)	44 (81.5)	REF 1.00	REF 1.00	7 (9.6)	44 (40.0)	REF 1.00	REF 1.00
Primary series, no booster dose	45 (73.8)	10 (18.5)	**0.25 (0.14–0.44)**	**0.28 (0.15, 0.56)**	46 (63.0)	51 (46.4)	**0.61 (0.49, 0.76)**	**0.54 (0.43, 0.69)**
Primary series plus ≥ 1 booster dose	NA	NA	NA	NA	20 (27.4)	15 (13.6)	**0.50 (0.33, 0.74)**	**0.44 (0.30, 0.66)**
Days since most recent vaccine dose								
14–89	7 (11.5)	2 (3.7)	REF 1.00	REF 1.00	16 (21.9)	10 (9.1)	REF 1.00	REF 1.00
90–179	9 (14.8)	3 (5.5)	1.13 (0.23–5.43)	0.70 (0.13, 3.86)	23 (31.5)	7 (6.4)	0.61 (0.27, 1.37)	0.66 (0.30, 1.44)
180–359	29 (47.5)	5 (9.3)	0.66 (0.15–2.89)	0.58 (0.13, 2.63)	18 (24.7)	17 (15.5)	1.26 (0.70, 2.29)	1.19 (0.69, 20.7)
≥ 360	0 (0.0)	0 (0.0)	NA	NA	9 (12.3)	32 (29.1)	**2.03 (1.21, 3.39)**	**1.82 (1.14, 2.90)**
Unvaccinated	16 (26.2)	44 (81.5)	3.30 (0.96–11.4)	2.40 (0.70, 8.28)	7 (9.6)	44 (40.0)	**2.24 (1.36, 3.70)**	**2.40 (1.52, 3.79)**

Booster doses were first available on Navajo Nation starting September 25, 2021, for adults aged ≥ 65 years, starting November 7, 2021, for those ages ≥ 18 who were at high risk for severe COVID-19, and starting November 10, 2021 for all persons aged ≥ 18 years. Results are stratified based on “booster eligibility,” determined by the date of illness onset per participant relative to their individual booster eligibility. Alaska sites only enrolled inpatients and over 90% of participants at the Whiteriver, AZ site were inpatients, so adult risk factor analysis restricted to Chinle and Tuba City sites only. Boldface indicates statistical significance of *p* < 0.05

*CI* 95% confidence interval, *NA* not applicable, *REF* reference category, *RR* risk ratio

^a^Separate adjusted models for vaccination status and days since vaccination. Models adjusted for age, service unit (enrollment site), chronic lung disease, chronic kidney disease, chronic liver disease, presence of diabetes, hypertension, and number of days between illness onset and presentation. All other covariates were not statistically significant in bivariable models and/or had zero participants in either the outpatient or inpatient category

^b^Vaccination status (unvaccinated, received primary series only, received primary series plus at least one booster dose) included in adjusted models. Completed primary series = Received two doses of an approved mRNA COVID-19 vaccine primary series or one dose of an approved non-mRNA vaccine ≥ 14 days prior to illness onset

In bivariable models, older age, chronic lung disease, chronic kidney disease, diabetes, home oxygen use prior to the illness event, and presenting for care eight days or more after illness onset were associated with increased risk for COVID-19-associated hospitalization (Table 3). Frequent mask wearing outside of the home (usually/always versus never/sometimes) was protective. In the multivariable model, older age (50–64 years adjusted risk ratio [aRR] = 1.44 [95% CI 1.13–1.85]; ≥ 65 years aRR = 1.53 [1.16–2.01]), chronic lung disease (aRR = 1.79 (1.07–3.01), chronic kidney disease (aRR = 1.67 [1.26–2.21]), and diabetes (aRR = 1.36 [1.10–1.68]) retained significance and were associated with increased risk for COVID-19-associated hospitalization; frequent mask use (aRR = 0.63 [0.49–0.81]) remained protective.

The impact of COVID-19 vaccination status and time since vaccination on COVID-19-associated hospitalization was stratified by availability of COVID-19 booster doses. Prior to COVID-19 vaccine availability, we enrolled 61 outpatients and 54 inpatients (Table 4). In bivariable and multivariable models, COVID-19 vaccination (i.e., having received a full primary series prior to illness onset) was significantly associated with reduced risk of hospitalization (aRR = 0.28 [0.15–0.56]), but the number of days since vaccination (14–89, 90–179, 180–359, or ≥ 360) was not (Table 4). After the availability of the monovalent booster dose, we enrolled 73 outpatients and 110 inpatients. In bivariable and multivariable models, completing a primary series only (aRR = 0.54 [0.43–0.69]) and completing a primary series and at least one booster dose (aRR = 0.44 [0.30–0.66]) were also associated with reduced risk of hospitalization (Table 4). Having been vaccinated ≥ 360 days ago (aRR = 1.82 [1.14–2.90]) and being unvaccinated (aRR = 2.40 [1.52–3.79]) were significantly associated with hospitalization compared to individuals who were vaccinated < 90 days ago. Similar results were found for severe COVID-19 associated hospitalization (Supplemental Table 2).

Among 625 participants aged < 18 years enrolled at the Chinle, Tuba City, and Whiteriver sites, 110 (17.6%) were SARS-CoV-2-positive, including 44 inpatients (40.0%) and 66 outpatients (60.0%). Median age was 4.5 years, 39.1% were female, 11.8% had asthma, and 19.1% had obesity (Table 5). Overall, 17 (15.5%) participants completed a primary COVID-19 vaccine series prior to their illness onset; of these, 14 (82.4%) received a primary series only and 3 (17.6%) received at least one booster dose. Of the 44 hospitalized children, 43 were unvaccinated (97.7%). Only two (4.5%) of the 44 hospitalizations were classified as severe. Few differences were found between consented, declined and excluded SARS-CoV-2-positive eligible patients < 18 years (Supplemental Table 4), with excluded individuals found to be significantly older than consented participants or those who declined enrollment. In the multivariable model, the presence of a heart condition (aRR = 2.00 [1.45–2.67]) was associated with increased risk of hospitalization and older age (5–17 years old) was associated with reduced risk (aRR = 0.56 [0.32–0.97]) (Table 5).

**Table 5:** Participant characteristics and risk factors for COVID-19-associated hospitalization among American Indian/Alaska Native children at participating facilities in Arizona, January 1, 2021–December 31, 2022

	Outpatient (*N* = 66)	Hospitalization^a^ (*N* = 44)	Crude RR (95% CI)	Adjusted RR (95% CI)^b^
Age in years (median [IQR])	8.0 (2.8–12.8)	1.7 (0.6–5.1)	**0.89 (0.84–0.95)**	Not included
Age group in years, *n* (%)				
0–4	27 (40.9)	33 (75.0)	REF 1.00	REF 1.00
5–17	39 (59.1)	11 (25.0)	**0.40 (0.23–0.71)**	**0.56 (0.32–0.97)**
Sex, *n* (%)				
Male	38 (57.6)	29 (65.9)	REF 1.00	Not included
Female	28 (42.4)	15 (34.1)	0.81 (0.49–1.32)	Not included
Medical condition or risk factor present, *n* (%)				
Asthma	7 (10.6)	6 (13.6)	1.18 (0.62–2.23)	Not included
Chronic lung disease	3 (4.5)	4 (9.1)	1.47 (0.74–2.93)	Not included
Chronic liver disease	0	0	NA	NA
Chronic kidney disease	3 (4.5)	0	0.00 (0.00–0.00)	Not included
Diabetes (type 1 or 2)	0	0	NA	NA
Heart condition	0	5 (11.4)	**2.69 (2.10–3.46)**	**2.00 (1.45–2.76)**
Hypertension	1 (1.5)	1 (2.3)	1.26 (0.31–5.15)	Not included
Immunocompromised	0	0	NA	NA
Mental health condition	0	0	NA	NA
Obesity	11 (16.7)	10 (22.7)	1.25 (0.74–2.10)	Not included
Supplemental oxygen use at home	0	2 (4.5)	**2.57 (2.03–3.26)**	1.29 (0.77–2.18)
Running water in home^c^, *n* (%)	50 (75.8)	40 (90.9)	2.00 (0.81–4.91)	Not included
Wood used to heat home, *n* (%)	41 (62.1)	33 (75.0)	1.46 (0.84–2.55)	Not included
Education level^c,d^, *n* (%)				
Some high school or less	5 (7.6)	4 (9.1)	Not included	Not included
High school diploma or GED	32 (48.5)	24 (54.5)	Not included	Not included
Some college or AA degree	21 (31.8)	10 (22.7)	Not included	Not included
College degree (incl. advanced)	2 (3.0)	2 (4.5)	Not included	Not included
Vaccination status, *n* (%)				
Unvaccinated	50 (75.8)	43 (97.7)	REF 1.00	REF 1.00
Completed primary series^e^	16 (24.2)	1 (2.3)	**0.13 (0.02–0.87)**	0.19 (0.03–1.38)
Days from illness onset to care seeking, *n* (%)				
0–7	65 (98.5)	43 (97.7)	REF 1.00	REF 1.00
≥ 8	1 (1.5)	1 (2.3)	1.26 (0.31–5.15)	Not included

Reference category for all medical conditions or risk factors, running water in home, and wood used to heat home was “no.” Boldface indicates statistical significance of *p* < 0.05. Alaska sites only enrolled inpatients, so child risk factor analysis restricted to Arizona sites only. Inpatients were screened 5–6 days per week and outpatients were screened 2–4 days per week

*AA* associate degree, *CI* 95% confidence interval, *GED* general educational development, *IQR* interquartile range, *NA* not applicable (i.e., zeros in cells), *RR* risk ratio, *REF* reference category

^a^Any hospitalization. Only two children classified as “severe”: one 1-year-old male and one 5-year-old female

^b^Adjusted for age group, presence of heart condition, supplemental oxygen use at home, and vaccination status

^c^Two (1.8%) participants missing running water in home and 10 (9.1%) missing education level

^d^Mother’s education

^e^Completed primary series = Received two doses of an approved mRNA COVID-19 vaccine primary series or one dose of an approved non-mRNA vaccine ≥ 14 days prior to illness onset. May or may not have received ≥ 1 booster dose

## Discussion

These data from a prospective, active surveillance system at eight healthcare facilities in Arizona and Alaska provide evidence that the incidence of COVID-19-associated hospitalization was high among AI/AN persons, with rates among children 0–4 years old twice that of national estimates. Severity of illness was strongly mitigated by COVID-19 vaccination among adults.

In our study, weekly COVID-19-associated hospitalization incidence rates peaked in January 2021, October 2021, January 2022, and November 2022, coinciding with the first winter surge and Delta and Omicron variant circulation and predominance, respectively. Hospitalization rates varied considerably by age group; across all sites, the highest rates were observed among adults aged ≥ 65 years. Rates also varied by site, with the highest peak and overall rates observed in Whiteriver, AZ, and the lowest observed in Anchorage, AK.

The timing of estimated peaks is consistent with those from other national and state-level COVID-19 hospitalization surveillance networks [29–32]. Contrary to estimates for AI/AN individuals from COVID-NET interactive dashboard for 2021 and 2022, age-specific annual incidence rates among AI/AN adults in this study were not markedly different from the US general population [32], suggesting a reduction in the disparity in burden of disease in these communities compared to the beginning of the pandemic, possibly from high coverage of the COVID-19 vaccine among adults [33]. In contrast, annual rates for children aged 0–4 years were over two times higher in this study than the general population, with a possible increase in disparity in 2022 compared to 2021.

While the CDC’s COVID-NET platform estimates national weekly COVID-19-associated hospitalization incidence using similar methods to those reported here, direct comparisons to this and other platforms (e.g., state health departments) are challenging due to methodologic differences. One main difference is that COVID-NET and some other platforms do not include symptoms as part of their case definitions [34]. Since COVID-19 often presents asymptomatically, platforms without symptom criteria are likely to overcount incidental COVID-19 cases (i.e., those admitted for other reasons but test positive for SARS-CoV-2) as primary cases (i.e., admitted specifically for COVID) [35]. Our screening methodology (i.e., identifying symptomatic ARI) excluded asymptomatic persons from incidence calculations. Therefore, results presented here provide more accurate estimates of primary COVID-19 hospitalizations among AI/AN persons, and the comparison with national estimates likely underestimates the magnitude of any disparity. In addition, differences in COVID-19 testing practices, choice of population denominators (e.g., census data, IHS User Population), and age groups further complicate comparisons. Despite limitations, these data suggest that control efforts in these communities succeeded in reducing the initial disparity in burden of disease among adults, but that disparities among children remained and warrant continued monitoring to inform prevention strategies [29–32].

Several other findings from this analysis warrant additional discussion. COVID-19-associated hospitalization incidence was notably higher in Whiteriver compared to other sites. Shortly after the White Mountain Apache Tribe issued an Emergency Declaration regarding the COVID-19 pandemic, IHS and community partners established a robust contact tracing and home visiting program, which included distribution of pulse oximeters for home use [36, 37]. The program’s highly successful community-based case identification and referral practices may have resulted in a higher proportion of appropriate referral to care, contributing to elevated rates. Conversely, the lowest rates were observed in Anchorage, the only urban site. This may be because many socioeconomic characteristics that may predispose persons to more severe health outcomes — including poverty, household crowding, challenges with accessing care, and health facility resource limitations — are less pronounced in urban areas [38, 39]. The high prevalence of comorbid conditions may have contributed to the disproportionate COVID-19-associated morbidity and mortality experienced by AI/AN persons early in the pandemic; however, even when underlying comorbidities are controlled for, one study from Mississippi found that the risk of death from COVID-19 was higher for AI/AN persons compared to Black and White Americans [40]. Improving health equity for AI/AN persons requires improvements in socioeconomic factors (e.g., housing conditions), continued investment to ensure accessibility and utilization of healthcare services, including vaccination, as well as interventions to address biological factors (e.g., comorbid conditions).

In the analysis of risk of COVID-19-associated hospitalization compared to COVID-19-associated outpatient visits among adults, COVID-19 vaccination was strongly associated with decreased risk of COVID-19-associated hospitalization, both before and after the availability of booster doses. These findings align with other studies indicating COVID-19 vaccination protects against severe COVID-19 outcomes, including hospitalization [41–44]. It has also been established that protection from COVID-19 vaccines wanes over time after vaccination, but to a lesser extent for serious disease compared to mild disease and infection [45, 46]. Consistent with these studies, our point estimates in the period after COVID-19 monovalent vaccine booster availability suggested greater risk of hospitalization for persons for whom it had been more than six months since their last vaccination, and results were statistically significant for persons for whom it had been more than one year since their last dose; this trend was not observed prior to booster availability [45, 46]. Overall, being unvaccinated was a stronger risk factor for COVID-19-associated hospitalization than the amount of time since vaccination. While the stratified analysis was limited by small sample sizes and some residual confounding cannot be ruled out, these results underscore the importance of maintaining robust vaccination efforts, especially among disproportionately affected groups such as AI/AN persons.

Older age and the presence of certain underlying medical conditions — such as chronic kidney disease and diabetes — were risk factors for hospitalization among adults [47]. One recent study demonstrated increased likelihood for severe COVID-19 outcomes among vaccinated persons aged ≥ 65 years (aOR = 3.22), persons with chronic kidney disease (aOR = 1.61), and persons with diabetes (aOR = 1.47), consistent with our findings [44]. Based on this research and the higher rates of some chronic underlying medical conditions among AI/AN persons compared to the general population [4, 5], interventions should be considered to ensure AI/AN people at increased risk are up to date with recommended COVID-19 vaccines, minimize exposure, and optimize chronic disease management.

The ability to evaluate risk factors among children was limited by the small number of SARS-CoV-2-positive children in our study. However, these results suggested that risk of hospitalization was two times greater among children with heart disease, consistent with previous findings [48–50]. Although obesity has been identified as a risk factor for severe COVID-19 disease among children [51–53], this comorbidity was not significant in multivariable analyses. Similar to adults, understanding the pathophysiological association between COVID-19 and underlying conditions (e.g., heart disease) is critical to informing interventions and reducing risk of severe disease among children. Although receipt of COVID-19 vaccination was not statistically significant after adjusting for other covariates, vaccine effectiveness estimates among children have demonstrated robust protection [54–56] and vaccination continues to be recommended to protect younger age groups from severe COVID-19 [57].

These results are subject to limitations. Study enrollment occurred at eight selected healthcare facilities in Arizona and Alaska, and results may not be generalizable to the 9.7 million people who identify as AI/AN in the USA, of whom 13% live on reservations or trust lands [6, 58]. Incidence rates were estimated using all eligible inpatients regardless of enrollment status, but it is possible eligible persons were missed during surveillance, which would lead to an underestimate of disease burden. The incidence analysis accounted for potential under-ascertainment of cases by weighting the numerator to impute the small proportion of missing SARS-CoV-2 test results; however, the analysis did not account for the added uncertainty from this approximation. Vaccination status was not available for non-enrolled patients resulting in a large number of persons for whom vaccination status would need to be imputed, and therefore we were unable to stratify incidence rate estimates by vaccination status. However, results of the risk factor analysis indicated that completion of a primary vaccine series (with or without monovalent booster) was associated with a reduced risk of severe disease, and it is likely that stratified incidence estimates would have yielded results consistent with these findings. The risk factor analysis was limited by the low consent acceptance among eligible inpatients included in the analyses (41.8% among all eligible persons and 41.3% among SARS-CoV-2-positive eligible persons), which was approximately 13 percentage points lower than other active surveillance systems [59]. Study staff were unable to consent more than half of eligible inpatients because these patients were transferred to higher-level facilities, were too sick during their hospitalization to approach, or declined enrollment. While the proportion testing positive for SARS-CoV-2 was similar among consented and non-consented individuals, SARS-CoV-2-positive consented patients were significantly younger and more were female than among the SARS-CoV-2-positive patients who were not consented. This limited our ability to assess risk factors for severe hospitalization and likely biased our characterization of COVID-19 hospitalizations towards younger patients and those associated with less severe disease [60]. In addition, participant interviews were conducted after the COVID-19 event (i.e., after hospitalization), and recall of potential risk factors — such as mask use — may be subject to recall bias.

## Conclusion

AI/AN communities in the USA have experienced substantial morbidity and mortality from the COVID-19 pandemic. Few sources provide data such as incidence rates of COVID-19-associated hospitalizations among AI/AN persons, and those that do likely underestimate true burden [13, 61]. Data about COVID-19 disease burden among AI/AN persons are needed to support Tribal communities in making evidence-based decisions to support the health and well-being of community members, advocating for resources for disease prevention, and developing public health education and outreach campaigns to reduce existing health inequities among AI/AN communities. Results from these analyses help address this gap and provide critically needed data regarding disease burden and risks for severe outcomes.

## Supplementary Information

Below is the link to the electronic supplementary material.

Supplementary file1 (DOCX 1303 KB)
