# Supplementary material for: Incidence Rates of COVID-19-Associated Hospitalization and Risk Factors for Severe Disease Among American Indian and Alaska Native Persons in the Southwest USA and Alaska
Source: J Racial Ethn Health Disparities. 2025 Jul 21;13(4):3205–19. doi: 10.1007/s40615-025-02492-9 (PMC13346257; doi:10.1007/s40615-025-02492-9)
Supplement: Supplementary file 1 — Supplementary file1 (DOCX 1303 KB) [file 40615_2025_2492_MOESM1_ESM.docx]

**Incidence rates of COVID-19-associated hospitalization and risk factors for severe disease among American Indian/Alaska Native persons in the Southwest United States and Alaska**

**SUPPLEMENTAL TABLES (4) AND FIGURES (11)**

| **Supplemental Table 1. Study definitions** | |
| --- | --- |
| Eligibility criteria for enrollment in the surveillance study | American Indian or Alaska Native person AND  At least one of: cough, shortness of breath, apnea (if ≤6 months), sepsis (if ≤6 months), asthma exacerbation, fever, chills, rigors, myalgia, headache, sore throat, coryza/congestion, new olfactory or gustatory disorder, clinical and/or radiological evidence of pneumonia, or clinical and/or radiological evidence of acute respiratory distress syndrome (ARDS) |
| COVID-19-associated hospitalization | Eligibility for surveillance study AND  Positive SARS-CoV-2 test within 14 days of the hospitalization |
| Vaccination status |  |
| Unvaccinated | Did not receive any doses of an approved mRNA COVID-19 vaccine at the time of illness onset, received one dose of an approved mRNA COVID-19 vaccine primary series ≥14 days prior to illness onset, received the second dose of an approved mRNA COVID-19 vaccine primary series 0 to 13 days prior to illness onset, or received a non-mRNA vaccine 0 to 13 days prior to illness onset. |
| Complete primary series (with or without booster) | Received two doses of an approved mRNA COVID-19 vaccine primary series or one dose of an approved non-mRNA vaccine ≥14 days prior to illness onset. May or may not have received ≥1 booster dose. |
| Complete primary series only | Received only two doses of an approved mRNA COVID-19 vaccine primary series or one dose of an approved non-mRNA vaccine ≥14 days prior to illness onset. Did not receive any booster dose. |
| Complete primary series plus ≥1 booster | Received two doses of an approved mRNA COVID-19 vaccine or one dose of an approved non-mRNA vaccine, plus at least one additional booster dose, ≥14 days prior to illness onset |

| **Supplementary Table 2. Enrollment sites** | | | | | |  |
| --- | --- | --- | --- | --- | --- | --- |
| **Facility Name** | **Location** | **Facility Description*** | **Population Served** | **Approximate Service Population** | **Surveillance Conducted** |  |
| **Chinle** | | | | | |  |
| Chinle Comprehensive Health Care Facility^1^ | Chinle Service Unit, Northeastern Navajo Nation, AZ | IHS facility  Hospital + smaller outpatient clinics; 60 hospital beds | Tribal members | 37,000 | Inpatient and outpatient |  |
| Pinon Health Center^2^ | Chinle Service Unit, Northeastern Navajo Nation, AZ | IHS facility  Outpatient clinic | Tribal members | 11,000 | Outpatient |  |
| **Tuba City** | | | | | |  |
| Tuba City Hospital^3,4^ | Tuba City Service Unit, Northwestern Navajo Nation, AZ | Tribal health facility  Hospital + outpatient clinics belonging to the Tuba City Regional Health Care Corporation; 73 hospital beds | Tribal members | 75,000 | Inpatient and outpatient |  |
| **Whiteriver** | | | | | |  |
| Whiteriver Indian Hospital^5^ | Whiteriver Service Unit, WMA Tribal lands, eastern AZ | IHS facility  Hospital + smaller outpatient clinics; 40 hospital beds | Tribal members | 17,000 | Inpatient |  |
| Cibecue Outpatient Center^5^ | Cibecue, AZ | IHS facility  Outpatient clinic (Whiteriver Hospital satellite clinic) | Tribal members | 17,000 | Outpatient |  |
| Summit Healthcare | Show Low, AZ | Not-for-profit healthcare facility  Outpatient clinic associated with Summit Healthcare Regional Medical Center; 101 hospital beds | Tribal members and non-tribal members (only Tribal members were enrolled) | 90,000 | Inpatient and Outpatient |  |
| **Anchorage** | | | | | |  |
| Alaska Native Medical Center^6^ | Anchorage, AK | Tribal health facility  Compact of 13 regional Tribal health corporations (Alaska Native Tribal Health Consortium); 173 hospital beds | Tribal members | 100,000 | Inpatient |  |
| **Yukon Kuskokwim Deta** | | | | | |  |
| Yukon Kuskokwim Delta Regional Hospital^7^ | Bethel, YK Delta, Southwestern AK | Tribally health facility  Inpatient hospital; 34 hospital beds | Tribal members | 23,000 | Inpatient |  |
| Abbreviations: AK: Alaska; AZ: Arizona; IHS, Indian Health Service; WMA, White Mountain Apache; YK: Yukon Kuskokwim  *For context, the Indian Health Service (IHS) and tribal health facilities provide free healthcare services to American Indian and Alaska Native individuals. Previous studies (not published) among the Southwest sites found that almost all individuals received care through IHS or tribal health facilities and only a small proportion sought care at a private facility. The sites included in this study are located in remote areas with limited access to health care facilities. | | | | | | |
| Table adapted from Lutz CS, et al (2023)^22^ Supplemental Material | | | | | | |
| 1. Indian Health Service. Chinle Comprehensive Health Care Facility. <https://www.ihs.gov/navajo/healthcarefacilities/chinle/>. Accessed 2022 Mar 15.  2. Indian Health Service. Pinon Health Center. <https://www.ihs.gov/navajo/healthcarefacilities/pinon/>. Accessed2022 Mar 15.  3. Tuba City Regional Health Care Corporation. *Annual Report 2020.* 2020.  4. Tuba City Regional Health Care Corporation. *Annual Report 2017.* 2017.  5. Indian Health Service. Whiteriver Indian Hospital. <https://www.ihs.gov/phoenix/healthcarefacilities/whiteriver/>. Accessed2022 Mar 15.  6. Alaska Native Tribal Health Consortium. Alaska Native Medical Center. <https://anthc.org/alaska-native-medical-center/>. Published 2022. Accessed 2022 Mar 15.  7. Yukon-Kuskokwim Health Corporation. About the YK Delta. [https://www.ykhc.org/story/about- yk/#:~:text=The%20Yukon%2DKuskokwim%20Delta%20region,communities%20not%20connected%20by%20road](https://www.ykhc.org/story/about-%20%20%20yk/#:~:text=The%20Yukon%2DKuskokwim%20Delta%20region,communities%20not%20connected%20by%20road). Published 2022. Accessed 2022 Mar 15. | | | | | | |

| **Supplemental Table 3. Participant characteristics and risk factors for severe COVID-19-associated hospitalization among American Indian/Alaska Native adults at participating facilities in Arizona, January 1, 2021 – December 31, 2022** | | | | |
| --- | --- | --- | --- | --- |
|  | **Outpatient (n=134)** | **Severe hospitalization (n=21)** | **Crude**  **RR (95% CI)** | **Adjusted**  **RR (95% CI)^a,b^** |
| **Age in years (median [IQR])** | 45.3 (33.0-60.3) | 53.8 (46.3-61.6) | 1.02 (1.00-1.04) | 1.05 (1.03-1.07) |
| **Age group in years** |  |  |  |  |
| 18–49 | 78 (58.2) | 8 (38.1) | REF 1.00 | REF 1.00 |
| 50–64 | 34 (25.4) | 8 (38.1) | 2.05 (0.82-5.09) | **2.69 (1.31-5.51)** |
| ≥65 | 22 (16.4) | 5 (23.8) | 1.99 (0.71-5.60) | **4.56 (2.27-9.16)** |
| **Sex** |  |  |  |  |
| Male | 36 (26.9) | 9 (42.9) | REF 1.00 | REF 1.00 |
| Female | 98 (73.1) | 12 (57.1) | 0.55 (0.25-1.21) | 0.62 (0.32-1.21) |
| **Medical condition or risk factor present** |  |  |  |  |
| Alcohol and/or substance abuse | 12 (9.0) | 5 (23.8) | **2.54 (1.06-6.06)** | 1.64 (0.67-4.03) |
| Asthma | 26 (19.4) | 3 (14.3) | 0.72 (0.23-2.30) | 0.79 (0.31-2.04) |
| Chronic lung disease | 1 (0.7) | 0 | NA | NA |
| Current or former smoker^b^ | 19 (14.2) | 2 (9.5) | 0.72 (0.18-2.89) | 0.77 (0.20-2.96) |
| Chronic liver disease | 7 (5.2) | 2 (9.5) | 1.71 (0.47-6.24) | **3.42 (1.23-9.48)** |
| Chronic kidney disease | 2 (1.5) | 1 (4.8) | 2.53 (0.48-13.28) | **2.02 (1.23-3.31)** |
| Diabetes (type 1 or 2) | 39 (29.1) | 10 (47.6) | 1.97 (0.89-4.33) | **2.40 (1.32-4.38)** |
| Heart condition | 18 (13.4) | 3 (14.3) | 1.06 (0.34-3.31) | 1.43 (0.54-3.78) |
| Hypertension | 37 (27.6) | 4 (19.0) | 0.65 (0.23-1.84) | 1.15 (0.48-2.75) |
| Immunocompromised | 4 (3.0) | 0 | NA | NA |
| Mental health condition | 18 (13.4) | 0 | NA | NA |
| Obesity | 38 (28.4) | 6 (28.6) | 1.01 (0.42-2.44) | 1.22 (0.59-2.50) |
| Supplemental oxygen use at home^c^ | 0 | 1 (4.8) | NA | NA |
| **Running water in home^c^** | 108 (80.6) | 13 (61.9) | 0.56 (0.22-1.44) | 0.77 (0.33-1.79) |
| **Wood used to heat home** | 77 (57.5) | 12 (57.1) | 0.99 (0.44-2.21) | 1.11 (0.56-2.18) |
| **Education level^c^** |  |  |  |  |
| Some high school or less | 15 (11.2) | 3 (14.3) | Not included | Not included |
| High school diploma or GED | 37 (27.6) | 4 (19.0) | Not included | Not included |
| Some college or AA degree | 56 (41.8) | 6 (28.6) | Not included | Not included |
| College degree (incl. advanced) | 20 (14.9) | 1 (4.8) | Not included | Not included |
| **Frequency of mask use outside of home^c^** |  |  |  |  |
| Never or sometimes | 4 (3.0) | 3 (14.3) | REF 1.00 | REF 1.00 |
| Usually or always | 126 (94.0) | 16 (76.2) | **0.26 (0.10-0.70)** | 0.40 (0.16-1.05) |
| **Days from illness onset to care seeking** |  |  |  |  |
| 0–7 | 122 (91.0) | 14 (66.7) | REF 1.00 | REF 1.00 |
| ≥8 | 12 (9.0) | 7 (33.3) | **3.58 (1.65-7.75)** | **2.59 (1.65-4.08)** |
| AA, associates degree; CI, 95% confidence interval; GED, general educational development; IQR, interquartile range; NA, not applicable (i.e., zeros in cells); REF, reference category; RR, risk ratio | | | | |
| Reference category for all medical conditions or risk factors, running water in home, and wood used to heat home was “no.” **Boldface** indicates statistical significance of p<0.05. | | | | |
| Alaska sites only enrolled inpatients and over 90% of participants at the Whiteriver, AZ site were inpatients, so adult risk factor analysis restricted to Chinle and Tuba City sites only. Inpatients were screened 5–6 days per week and outpatients were screened 2–4 days per week. | | | | |
| ^a^Adjusted for vaccination status only. | | | | |
| ^b^ Vaccination status (unvaccinated, received primary series only, received primary series plus at least one booster dose) included in adjusted model. Completed primary series = Received two doses of an approved mRNA COVID-19 vaccine primary series or one dose of an approved non-mRNA vaccine ≥14 days prior to illness onset. | | | | |
| ^c^ 6 (3.9%) participants missing current or former smoking status, 1 (0.7%) missing supplemental oxygen use at home, 8 (5.2%) missing running water in home, 13 (8.4%) missing education level, 6 (3.9%) missing frequency of mask use outside of home. Variables with >10% missingness were not included in univariable or multivariable models. | | | | |

| **Supplemental Table 4. Differences in select demographic characteristics between consented, excluded, and declined eligible American Indian/Alaska Native inpatients positive for SARS-CoV-2 from sites included in risk factor analyses, January 1, 2021 – December 31, 2022** | | | | | |
| --- | --- | --- | --- | --- | --- |
|  | **Consented** | **Declined** | **Excluded** |  | **Total** |
|  | **N (%)** | **N (%)** | **N (%)** | **P-value*** | **N (%)** |
| **Eligible Adults (≥18 years)** | | | | | |
| Total | 164 (35.8%) | 134 (29.3%) | 160 (34.9%) |  | 458 (100.0%) |
| Age group, y |  |  |  |  |  |
| 18–49 | 55 (33.5%) | 42 (31.3%) | 30 (18.8%) | <0.01 | 127 (27.7%) |
| 50–64 | 62 (37.8%) | 31 (23.1%) | 33 (20.6%) |  | 126 (27.5%) |
| ≥65 | 47 (28.7%) | 61 (45.5%) | 97 (60.6%) |  | 205 (44.8%) |
| Enrollment site |  |  |  |  |  |
| Chinle, AZ | 92 (56.1%) | 90 (67.2%) | 117 (73.1%) | 0.01 | 299 (65.3%) |
| Tuba City, AZ | 72 (43.9%) | 44 (32.8%) | 43 (26.9%) |  | 159 (34.7%) |
| Sex |  |  |  |  |  |
| Male | 58 (35.4%) | 67 (50.0%) | 68 (42.5%) | 0.04 | 193 (42.1%) |
| Female | 106 (64.6%) | 67 (50.0%) | 92 (57.5%) |  | 265 (57.9%) |
| **Eligible Children (<18 years)** | | | | | |
| Total | 44 (60.3%) | 18 (24.7%) | 11 (15.1%) |  | 73 (100.0%) |
| Age group, y |  |  |  |  |  |
| 0–4 | 33 (75.0%) | 16 (88.9%) | 6 (54.5%) | 0.14 | 55 (75.3%) |
| 5–17 | 11 (25.0%) | 2 (11.1%) | 5 (45.5%) |  | 18 (24.7%) |
| Enrollment site |  |  |  |  |  |
| Chinle, AZ | 19 (43.2%) | 12 (66.7%) | 5 (45.5%) | 0.19 | 36 (49.3%) |
| Tuba City, AZ | 2 (4.5%) | 1 (5.6%) | 2 (18.2%) |  | 5 (6.8%) |
| Whiteriver, AZ | 23 (52.3%) | 5 (27.8%) | 4 (36.4%) |  | 32 (43.8%) |
| Sex |  |  |  |  |  |
| Male | 29 (65.9%) | 13 (72.2%) | 6 (54.5%) | 0.62 | 48 (65.8%) |
| Female | 15 (34.1%) | 5 (27.8%) | 5 (45.5%) |  | 25 (34.2%) |

*P-values calculated using Pearson’s chi-square or Fisher’s exact test as appropriate.

| **Supplemental** **Figure 1. Weekly incidence rates of COVID-19-associated hospitalizations, by age group, among American Indian/Alaska Native persons in Anchorage, AK, January 1, 2021–December 31, 2022 (using the 3-week moving average)** |  |
| --- | --- |
| 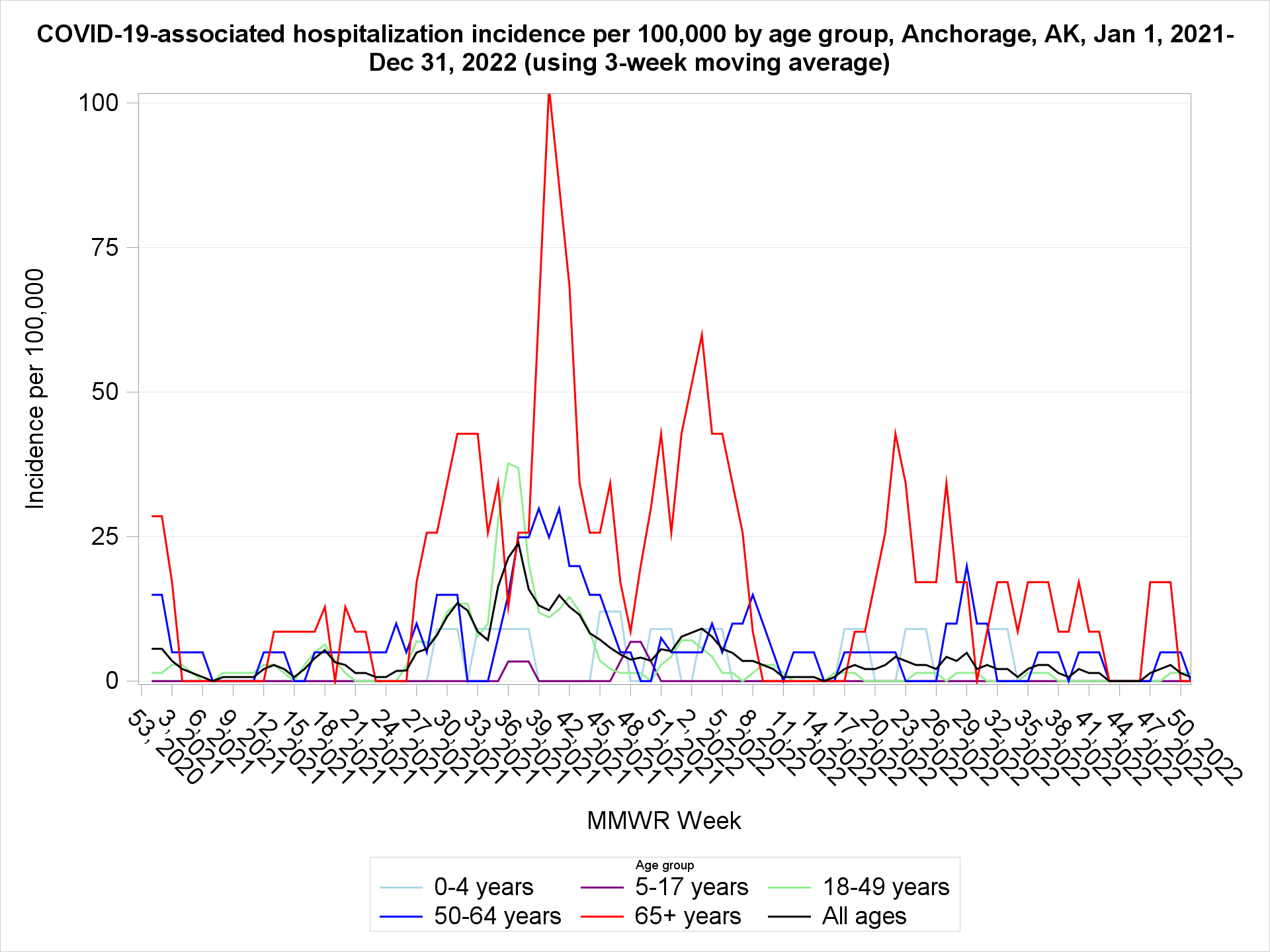 |  |
| MMWR Week 53, 2020 corresponds to the week ending 01/02/2021; MMWR Week 52, 2021 corresponds to the week ending 01/01/2022; MMWR Week 52, 2022 corresponds to the week ending December 31, 2022. MMWR weeks start on Sunday and end on the following Saturday. |  |
| **Supplemental** **Figure 2. Weekly incidence rates of COVID-19-associated hospitalizations, by age group, among American Indian/Alaska Native persons in Yukon-Kuskokwim Delta, AK, January 1, 2021–December 31, 2022 (using the 3-week moving average)** | |
| 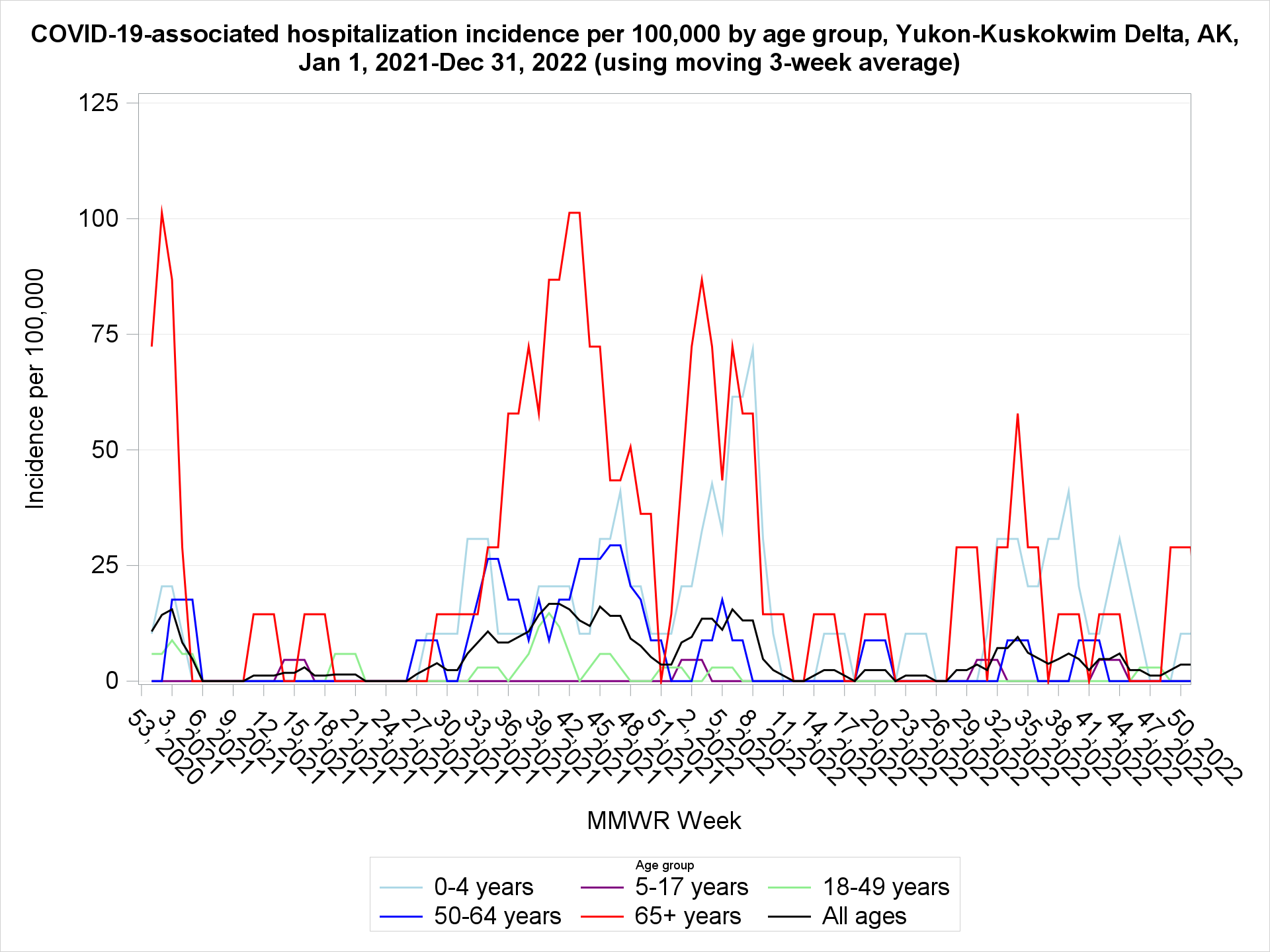 | |
| MMWR Week 53, 2020 corresponds to the week ending 01/02/2021; MMWR Week 52, 2021 corresponds to the week ending 01/01/2022; MMWR Week 52, 2022 corresponds to the week ending December 31, 2022. MMWR weeks start on Sunday and end on the following Saturday. | |

| **Supplemental** **Figure 3. Weekly incidence rates of COVID-19-associated hospitalizations, by age group, among American Indian/Alaska Native persons in Chinle, AZ, January 1, 2021–December 31, 2022 (using the 3-week moving average)** |
| --- |
| 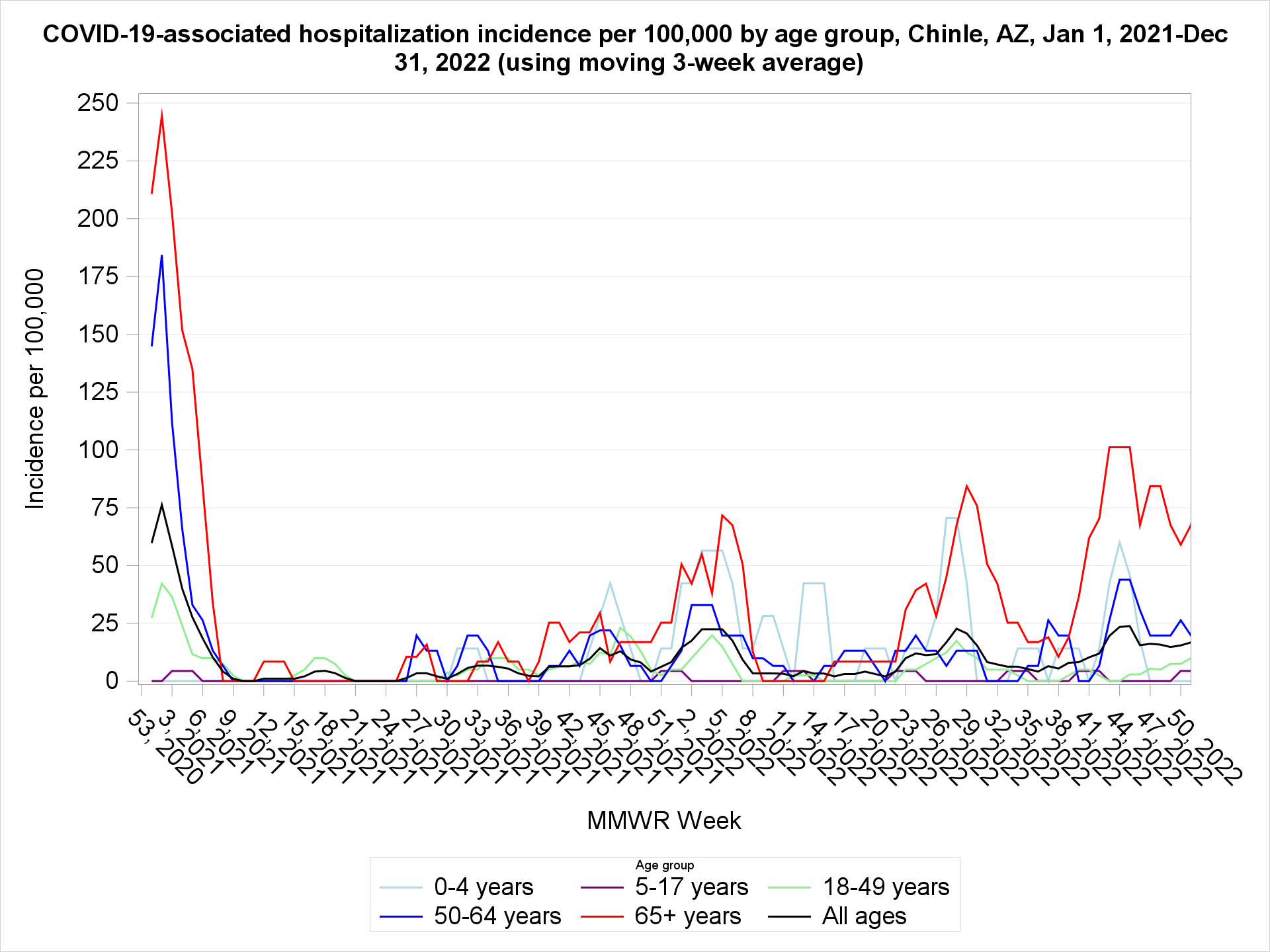 |
| MMWR Week 53, 2020 corresponds to the week ending 01/02/2021; MMWR Week 52, 2021 corresponds to the week ending 01/01/2022; MMWR Week 52, 2022 corresponds to the week ending December 31, 2022. MMWR weeks start on Sunday and end on the following Saturday. |

| **Supplemental** **Figure 4. Weekly incidence rates of COVID-19-associated hospitalizations, by age group, among American Indian/Alaska Native persons in Tuba City, AZ, January 1, 2021–December 31, 2022 (using the 3-week moving average)** |
| --- |
| 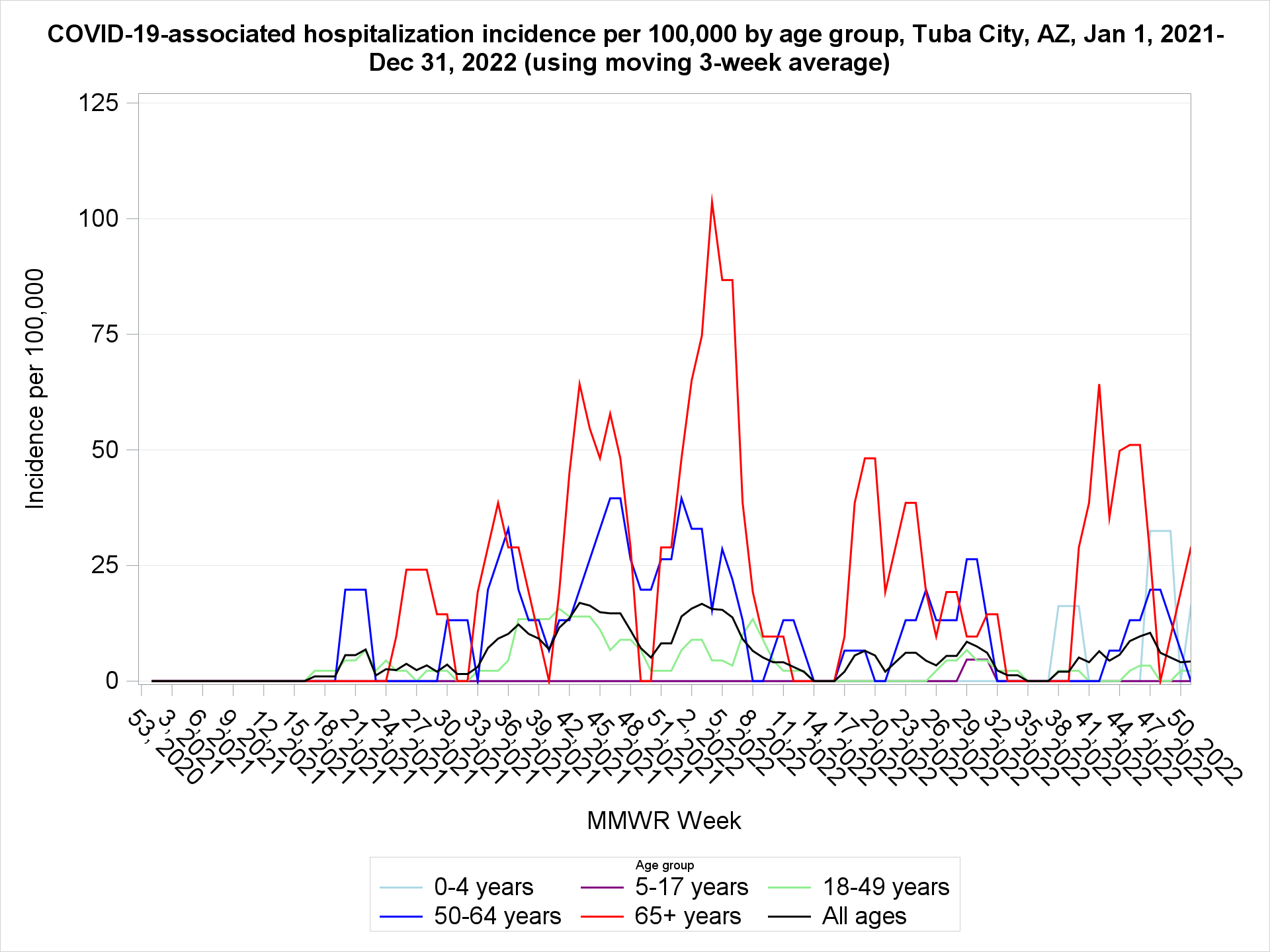  Tuba City began enrollment on May 17, 2021 |
| MMWR Week 53, 2020 corresponds to the week ending 01/02/2021; MMWR Week 52, 2021 corresponds to the week ending 01/01/2022; MMWR Week 52, 2022 corresponds to the week ending December 31, 2022. MMWR weeks start on Sunday and end on the following Saturday. |

| **Supplemental** **Figure 5. Weekly incidence rates of COVID-19-associated hospitalizations, by age group, among American Indian/Alaska Native persons in Whiteriver, AZ, January 1, 2021–December 31, 2022 (using the 3-week moving average)** |
| --- |
| 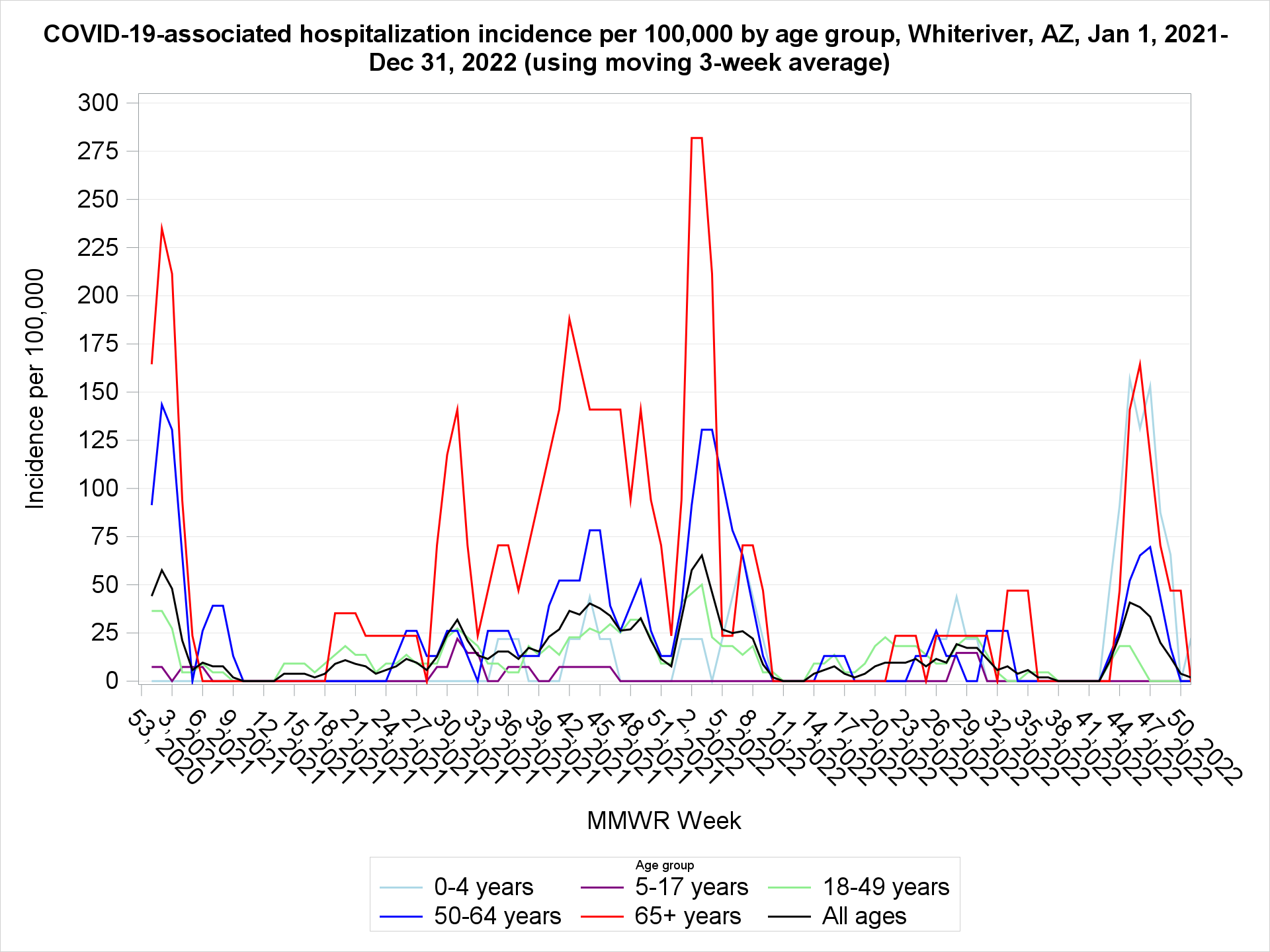 |
| MMWR Week 53, 2020 corresponds to the week ending 01/02/2021; MMWR Week 52, 2021 corresponds to the week ending 01/01/2022; MMWR Week 52, 2022 corresponds to the week ending December 31, 2022. MMWR weeks start on Sunday and end on the following Saturday. |

| **Supplemental** **Figure 6. Weekly incidence rates of COVID-19-associated hospitalizations among American Indian/Alaska Native persons at participating facilities in Arizona and Alaska, by site, all ages combined, January 1, 2021–December 31, 2022 (using the 3-week moving average)** |
| --- |
| 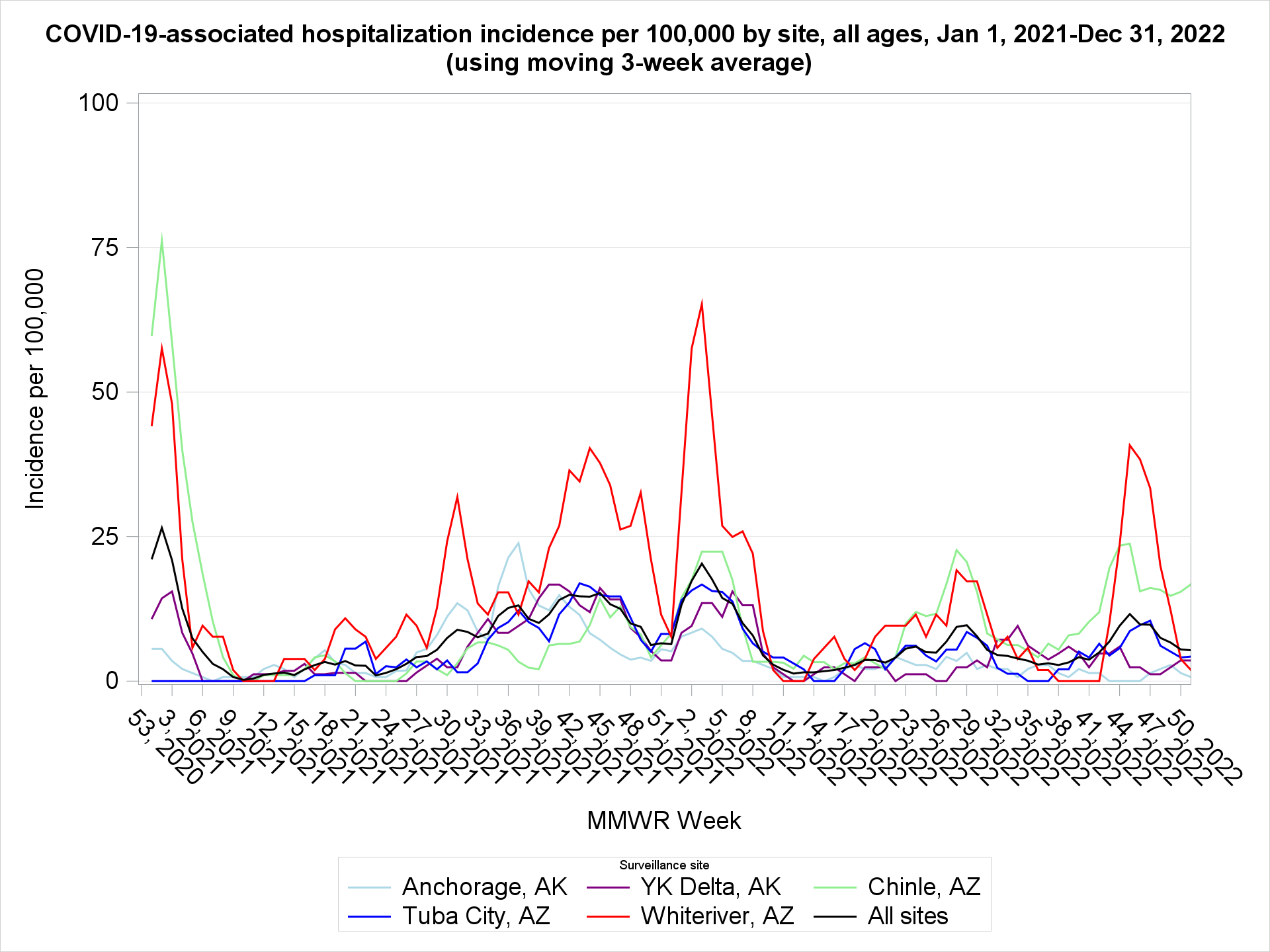 |
| MMWR Week 53, 2020 corresponds to the week ending 01/02/2021; MMWR Week 52, 2021 corresponds to the week ending 01/01/2022; MMWR Week 52, 2022 corresponds to the week ending December 31, 2022. MMWR weeks start on Sunday and end on the following Saturday. |

| **Supplemental Figure 7. Annual incidence rates of COVID-19-associated hospitalizations among American Indian/Alaska Native children 0 to 4 years old at participating facilities in Arizona and Alaska, by site, January 1, 2021–December 31, 2022** |
| --- |
|  |
| Tuba City 2021 denominator adjusted to account for only 33 weeks of surveillance. |

| **Supplemental Figure 8. Annual incidence rates of COVID-19-associated hospitalizations among American Indian/Alaska Native children 5–17 years old at participating facilities in Arizona and Alaska, by site, January 1, 2021–December 31, 2022** |
| --- |
|  |
| Tuba City 2021 denominator adjusted to account for only 33 weeks of surveillance. |

| **Supplemental Figure 9. Annual incidence rates of COVID-19-associated hospitalizations among American Indian/Alaska Native adults 18–49 years old at participating facilities in Arizona and Alaska, by site, January 1, 2021–December 31, 2022** |
| --- |
|  |
| Tuba City 2021 denominator adjusted to account for only 33 weeks of surveillance. |

| **Supplemental Figure 10. Annual incidence rates of COVID-19-associated hospitalizations among American Indian/Alaska Native adults 50–64 years old at participating facilities in Arizona and Alaska, by site, January 1, 2021–December 31, 2022** |
| --- |
|  |
| Tuba City 2021 denominator adjusted to account for only 33 weeks of surveillance. |

| **Supplemental Figure 11. Annual incidence rates of COVID-19-associated hospitalizations among American Indian/Alaska Native adults ≥65 years old at participating facilities in Arizona and Alaska, by site, January 1, 2021–December 31, 2022** |
| --- |
|  |
| Tuba City 2021 denominator adjusted to account for only 33 weeks of surveillance. |
